# Cross-Receiver Radio Frequency Fingerprint Identification: A Source-Free Adaptation Approach

**DOI:** 10.3390/s25144451

**Published:** 2025-07-17

**Authors:** Jian Yang, Shaoxian Zhu, Zhongyi Wen, Qiang Li

**Affiliations:** 1 School of Cyberspace Science and Technology, Beijing Institute of Technology, Beijing 100080, China; 2 School of Information and Communication Engineering, University of Electronic Science and Technology of China, Chengdu 611731, China

**Keywords:** radio frequency fingerprinting identification, source-free unsupervised domain adaptation, cross-receiver RFFI

## Abstract

Radio frequency fingerprint identification (RFFI) leverages the unique characteristics of radio signals resulting from inherent hardware imperfections for identification, making it essential for applications in telecommunications, cybersecurity, and surveillance. Despite the advancements brought by deep learning in enhancing RFFI accuracy, challenges persist in model deployment, particularly when transferring RFFI models across different receivers. Variations in receiver hardware can lead to significant performance declines due to shifts in data distribution. This paper introduces the source-free cross-receiver RFFI (SCRFFI) problem, which centers on adapting pre-trained RF fingerprinting models to new receivers without needing access to original training data from other devices, addressing concerns of data privacy and transmission limitations. We propose a novel approach called contrastive source-free cross-receiver network (CSCNet), which employs contrastive learning to facilitate model adaptation using only unlabeled data from the deployed receiver. By incorporating a three-pronged loss function strategy—minimizing information entropy loss, implementing pseudo-label self-supervised loss, and leveraging contrastive learning loss—CSCNet effectively captures the relationships between signal samples, enhancing recognition accuracy and robustness, thereby directly mitigating the impact of receiver variations and the absence of source data. Our theoretical analysis provides a solid foundation for the generalization performance of SCRFFI, which is corroborated by extensive experiments on real-world datasets, where under realistic noise and channel conditions, that CSCNet significantly improves recognition accuracy and robustness, achieving an average improvement of at least 13% over existing methods and, notably, a 47% increase in specific challenging cross-receiver adaptation tasks.

## 1. Introduction

**Figure 1:**
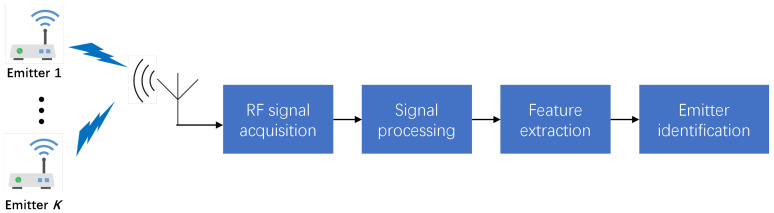
The block diagram of the RFFI system outlines the following sequence: The process begins with the emission of signals from multiple sources. These signals are captured by a receiver, after which they undergo signal processing. This step typically includes operations such as filtering, amplification, and the conversion of the signals from analog to digital form. Once processed, the system proceeds to the feature extraction and identification phase, where distinctive characteristics or “fingerprints” of the signals are identified.

Radio frequency fingerprint identification (RFFI) is an emerging technology that leverages the unique characteristics of radio signals emitted by devices for identification purposes. It is based on the concept that every electronic device, due to hardware imperfections introduced during the manufacturing process, emits a unique and repeatable RF signal, often referred to as a radio frequency fingerprint [1]. These unique signatures are crucial for applications in secure communication systems, device authentication, and surveillance. The integration of deep learning (DL) has significantly improved the accuracy of RFFI systems, which typically consist of RF signal acquisition, feature extraction, and emitter identification stages, as illustrated in Figure 1. Despite these advancements, a critical challenge for practical deployment remains: The performance of a DL-based model degrades significantly when it is transferred from the receiver on which it was trained to a new one. This is known as the *Cross-receiver RFFI* problem, caused by data distribution shifts arising from variations in receiver hardware characteristics [2,3].

While existing methods have made progress in improving cross-receiver generalization, they almost universally assume that data from the source domain are available during the adaptation phase. However, in many practical applications, this requirement is challenging to meet. Privacy concerns, security protocols, and the logistical difficulties of transmitting large volumes of raw RF data often make accessing original training data from other receivers impractical. Therefore, a more feasible and secure approach is to adapt an RFFI model to a new receiver without accessing the original training data, relying only on the pre-trained model itself. Our research is driven by the need to address this gap. This paper defines and tackles this new, more stringent problem, which we term *source-free cross-receiver RFFI* (SCRFFI). This framework sets our work apart from existing methods that depend on source data [2,3,4,5,6,7,8,9,10,11,12].

The cross-receiver RFFI problem has been extensively studied from a domain adaptation perspective. Researchers have explored various paradigms, such as training with data from multiple receivers to enhance generalization [2], or developing calibration procedures to align signals from new receivers [2]. Other prominent approaches include adversarial training to learn receiver-agnostic features [3,9,12], and feature disentanglement to separate transmitter characteristics from receiver-induced effects [5,11]. More recent works have advanced these ideas using dynamic distribution alignment with maximum mean discrepancy (MMD) [8], prototypical contrastive domain adaptation learning (PCDAL) [10], and hybrid strategies for specific applications like UAV RFFI [12]. Nonetheless, all these techniques necessitate access to source domain data, making them unsuitable for the SCRFFI scenario we address.

To solve the challenging SCRFFI problem, where source data is inaccessible, we propose a novel method called the contrastive source-free cross-receiver network (CSCNet). Our approach begins by introducing pseudo-labeling and providing a theoretical analysis of its generalization performance on the new receiver. CSCNet employs contrastive learning alongside a three-pronged loss function—combining information entropy minimization, a pseudo-label self-supervised loss, and a contrastive learning loss—to effectively adapt the model to the new receiver’s characteristics using only its unlabeled data. This comprehensive approach directly addresses the performance degradation due to receiver hardware variations by learning robust, domain-invariant features and inherently overcomes the limitation of requiring access to original source training data.

The main contributions of this paper are summarized as follows:

- We define and address the SCRFFI problem, which centers on adapting a trained RF fingerprinting model to new receivers without requiring access to the original labeled data from other receivers. This approach is particularly suited for real-world applications where data privacy and transmission constraints are critical concerns.
- We introduce the concept of pseudo-labeling and formulate a novel learning problem specific to SCRFFI. Through rigorous analysis, we derive an upper bound on the generalization performance of the proposed solution, establishing a solid theoretical foundation for our approach.
- To address the SCRFFI problem, we propose CSCNet, an innovative method that incorporates contrastive learning. CSCNet employs a three-pronged loss function strategy, allowing the model to emphasize the relationships between signal samples and effectively adapt to the characteristics of new receivers using only unlabeled test data.
- Experiments on two real-world datasets demonstrate that CSCNet outperforms existing methods in terms of recognition accuracy and robustness.

The remainder of the paper is organized as follows. Section 2 introduces the system model and defines the SCRFFI problem; Section 3 performs a theoretical analysis on the SCRFFI problem; Section 4 presents the design of the proposed CSCNet; Section 5 evaluates the performance of CSCNet on HackRF and Wisig datasets; and Section 6 concludes the paper.

**Notation 1.**  *∥·∥ denotes $\ell_{2}$ norm; 〈·,·〉 represents the inner product, ${\left[a\right]}_{k}$ denotes the k-th entry of the vector a, $1_{a\neq b}$ is an indicator function whose value equals one if a≠b, and 0 otherwise; $\Delta_{K}$ denotes the (K−1)-dimension probability simplex, i.e., $\Delta_{K}=\left\{z\in R^{K}\;|\;z_{k}\;\geq \;0,k\;=\;1,2,\ldots ,K,\sum_{k=1}^{K}z_{k}=1\right\}$; ⊛ denotes the convolution operator; E[·] denotes the expectation.*

## 2. System Model and Problem Formulation

### 2.1. System Model

During the *i*-th time interval, the RF signal received, denoted as $x^{i}\left(t\right)$, is modeled by the equation

$$x^{i}\left(t\right)=\zeta \left(c\left(t\right)⊛\nu \left(s\left(t\right)\cos\left(2\pi f_{0}t+\phi \right)\right)\right)+n\left(t\right),$$

for (i−1)T≤t≤iT. In this model, n(t) signifies noise, *T* is the duration of the interval, $f_{0}$ is the carrier frequency, s(t) is a random modulating signal, and c(t) represents the channel response. The function ν(·) accounts for nonlinear distortions caused by the emitter’s hardware, while ζ(·) addresses the nonlinear reception characteristics inherent to the receiver’s hardware. It is important to note that each receiver generally has a unique ζ.

In the above model, the nonlinear distortion function ν is essential for capturing the unique RF fingerprint of an emitter. This function reflects the hardware imperfections inherent to the emitting device, which arise from various sources during signal generation and transmission, such as amplifiers and mixers. For instance, power amplifiers often exhibit nonlinear behavior when driven beyond their linear operating range, resulting in the following relationship:

$$\tilde{y}\left(t\right)=\alpha_{1}\tilde{x}\left(t\right)+\alpha_{2}{\tilde{x}}^{2}\left(t\right)+\alpha_{3}{\tilde{x}}^{3}\left(t\right)+\ldots$$

Here, $\tilde{y}\left(t\right)$ is the output signal, $\tilde{x}\left(t\right)$ is the input signal, and $\alpha_{n}$ are the coefficients that characterize the degree of nonlinearity. When $\alpha_{2}$ significantly exceeds $\alpha_{1}$, the output exhibits notable distortion, which can manifest as harmonic generation or intermodulation effects. Such distortions introduce unwanted harmonic frequency components, affecting signal integrity and contributing to the emitter’s unique fingerprint. Additionally, the specific design of the transmitter’s circuitry can introduce further nonlinear effects. Components such as filters, oscillators, and even the layout of the circuit board can influence how the signal is processed and distorted. Given the various sources of distortion contributing to the emitter’s RF fingerprint, accurately modeling it with a simple function is challenging. However, leveraging the powerful modeling capabilities of deep neural networks allows us to adopt a data-driven approach to automatically capture the distinct distortions among emitters.

For simplicity, we define $x_{s}^{i}$ as the *i*-th processed signal sample and $y_{s}^{i}\in K≜\left\{1,\ldots ,K\right\}$ as the corresponding emitter label, where *K* denotes the number of emitters and the subscript *s* indicates the source domain $D_{s}$ for Rx-1. Thus, the labeled training dataset for Rx-1 is represented concisely as $D_{s}={\left\{(x_{s}^{i},y_{s}^{i})\right\}}_{i=1}^{N_{s}}$, with $N_{s}$ being the dataset size. In a similar fashion, the dataset from a new receiver, denoted by Rx-2, is represented as $D_{t}={\left\{x_{t}^{i}\right\}}_{i=1}^{N_{t}}$, where the subscript *t* denotes the target domain associated with Rx-2. Notice that the target dataset $D_{t}$ has no label information. We should mention that the subsequent developed approach does not hinge on the number of source receivers. Thus, for ease of exposition, we consider one source receiver; extension to multiple source receivers is straightforward.

### 2.2. Source-Free Cross-Receiver RFFI

The problem of SCRFFI can be described as follows: Let $h_{s}:D_{s}\to \Delta_{K}$ denote the model trained on labeled data from the source domain $D_{s}$. With $h_{s}$ known and utilizing unlabeled data from the target domain $D_{t}$, our objective is to develop an RFFI model $h_{t}:D_{t}\to \Delta_{K}$ that minimizes the classification error on $D_{t}$. Specifically, our goal is to minimize

(1) $$\begin{array}{cc} \; & \min_{h_{t}\in H}\epsilon (h_{t},f_{t})≜E_{X_{t}∼D_{t}}\left[1_{\arg\;\max\left(h_{t}\left(X_{t}\right)\right)\neq \arg\;\max\left(f_{t}\left(X_{t}\right)\right)}\right]\\ \approx & \min_{h_{t}\in H}\hat{\epsilon }\left(h_{t},f_{t}\right)≜\frac{1}{N_{t}}\sum_{x_{t}^{i}\in D_{t}}1_{\arg\;\max\left(h_{t}\left(x_{t}^{i}\right)\right)\neq y_{t}^{i}}, \end{array}$$

where $f_{t}:D_{t}\to \Delta_{K}$ represents the unknown true label function for the target domain. Here, $\epsilon (h,f_{t})$ and $\hat{\epsilon }\left(h,f_{t}\right)$ denote the expected risk and empirical risk, respectively, when applying a classification model *h* to the target domain. The hypothesis space H encompasses all potential models mapping from $D_{t}$ to $\Delta_{K}$. The specific form and capacity of H are dictated by the architecture and size of the neural network employed. The efficacy of the classification model is closely tied to the complexity of H, often characterized by metrics such as the Vapnik–Chervonenkis (VC) dimension [13]. This will be further discussed in the subsequent section.

## 3. Theoretical Analysis

To gain insights into our proposed design, we begin with a theoretical analysis of the SCRFFI problem. Directly minimizing problem (1) is not possible due to the lack of information about $f_{t}$. Therefore, we turn to the pseudo-label technique and define a pseudo-label function ${\tilde{f}}_{t}:D_{t}\to \Delta_{K}$ for the target domain. An example of such a function could be the original source model $h_{s}$. The next section will detail how to improve ${\tilde{f}}_{t}$ using $h_{s}$ and $D_{t}$. For the purpose of this analysis, we assume that an ${\tilde{f}}_{t}$ is already available for model adaptation. To simplify our discussion, we focus on the case where K=2. Now, let us consider the following learning problem in the target domain:

(2a) $$\begin{array}{cc} \min_{h\in H} & \;\hat{\epsilon }\left(h,{\tilde{f}}_{t}\right) \end{array}$$

(2b) $$\begin{array}{cc} s.t. & \;L^{\mathrm{entropy}}\left(h\right)\leq \rho , \end{array}$$

where ρ>0 is a small number close to zero, and

(3) $$L^{\mathrm{entropy}}\left(h\right)≜-\frac{1}{N_{t}}\sum_{i=1}^{N_{t}}\sum_{k=1}^{K}{\left[h\left(x_{t}^{i}\right)\right]}_{k}\cdot \log{\left[h\left(x_{t}^{i}\right)\right]}_{k}$$

with ${\left[h\right]}_{k}$ being the *k*-th entry of the vector *h*.

The problem in (2) serves as a manageable approximation for the problem in (1), aiming to minimize empirical risk relative to ${\tilde{f}}_{t}$ while incorporating additional constraints. Because ${\tilde{f}}_{t}$ is generally imprecise (i.e. ${\tilde{f}}_{t}\neq f_{t}$), merely minimizing $\hat{\epsilon }\left(h,{\tilde{f}}_{t}\right)$ is inadequate for enhancing the model’s performance. However, as the following theorem demonstrates, constraint (2b) plays a crucial role in facilitating effective model learning on the target domain.

**Theorem 1.**  *Let $\hat{h}$ be an optimal solution of problem* (2)*. Suppose that the hypothesis space H has a VC dimension d. Then, for any ρ∈(0,1), with probability at least 1−ρ, the following inequality holds:*

(4) $$\epsilon \left(\hat{h},f_{t}\right)\leq 2\epsilon \left({\tilde{f}}_{t},f_{t}\right)+c_{1}$$

*where $c_{1}=2\sqrt{\frac{d\left(\log\left(2N_{t}/d\right)+1\right)+\log\left(4/\rho \right)}{N_{t}}}$.*

**Proof of Theorem 1.**  First, we show that the ground-truth label function $f_{t}$ is a feasible solution to problem (2). By the definition of $f_{t}$, we know $f_{t}\left(x_{t}\right)\in \Delta_{K}$ is a one-hot vector for all $x_{t}\in D_{t}$. By substituting $f_{t}$ into (3), we have $L^{\mathrm{entropy}}\left(f_{t}\right)=0$, which satisfies the constraint (2b). Therefore, $f_{t}$ is a feasible solution of problem (2).

Next, according to the VC theory [13], the expected loss $\epsilon (\hat{h},f_{t})$ can be bounded by its empirical estimate $\hat{\epsilon }\left(\hat{h},f_{t}\right)$. Specifically, if $D_{t}$ is an $N_{t}$-sized i.i.d. sample, then, with probability exceeding 1−ρ,

$$|\epsilon \left(\hat{h},f_{t}\right)-\hat{\epsilon }\left(\hat{h},f_{t}\right)|\leq c_{1}/2.$$

Therefore, we have

(5) $$\epsilon \left(\hat{h},f_{t}\right)\leq \hat{\epsilon }\left(\hat{h},f_{t}\right)+c_{1}/2.$$

Then, it follows from the triangle inequality [14], i.e.,

$$\hat{\epsilon }\left(\hat{h},f_{t}\right)\leq \hat{\epsilon }\left(\hat{h},{\tilde{f}}_{t}\right)+\hat{\epsilon }\left({\tilde{f}}_{t},f_{t}\right)$$

that the right-hand side of (5) can be further bounded as

(6) $$\hat{\epsilon }\left(\hat{h},f_{t}\right)\leq \hat{\epsilon }\left(\hat{h},{\tilde{f}}_{t}\right)+\hat{\epsilon }\left({\tilde{f}}_{t},f_{t}\right)+c_{1}/2.$$

Combining (5) and (6) yields

(7) $$\epsilon \left(\hat{h},f_{t}\right)\leq \hat{\epsilon }\left(\hat{h},{\tilde{f}}_{t}\right)+\hat{\epsilon }\left({\tilde{f}}_{t},f_{t}\right)+c_{1}/2.$$

Since $\hat{h}$ is an optimal solution of problem (2), we have

$$\hat{\epsilon }\left(\hat{h},{\tilde{f}}_{t}\right)\leq \hat{\epsilon }\left(f_{t},{\tilde{f}}_{t}\right)$$

which, together with (7), implies

(8) $$\epsilon \left(\hat{h},f_{t}\right)\leq 2\hat{\epsilon }\left(f_{t},{\tilde{f}}_{t}\right)+c_{1}/2.$$

Finally, applying the VC theory [13] again to ${\hat{\epsilon }}_{t}\left(f_{t},{\tilde{f}}_{t}\right)$, we get

$$|\hat{\epsilon }\left(f_{t},{\tilde{f}}_{t}\right)-\epsilon \left(f_{t},{\tilde{f}}_{t}\right)|\leq c_{1}/2.$$

Substituting the above inequality into (8) yields

$$\epsilon \left(\hat{h},f_{t}\right)\leq 2\epsilon \left(f_{t},{\tilde{f}}_{t}\right)+c_{1}.$$

This completes the proof. □

The inequality presented in (4) highlights that the expected classification error probability for $\hat{h}$ on the target domain $D_{t}$ is constrained by a certain constant. This constant is influenced by the number of samples $N_{t}$, the capacity *d* of the hypothesis space H, and the precision of the pseudo-label function ${\tilde{f}}_{t}$. Notably, $c_{1}$ is typically a fixed value that does not depend on the learning method used. Consequently, enhancing the accuracy of the pseudo-label function ${\tilde{f}}_{t}$ is crucial for minimizing this upper bound. If ${\tilde{f}}_{t}$ perfectly matches $f_{t}$, then $\epsilon ({\tilde{f}}_{t},f_{t})$ approaches zero.

## 4. Proposed Method

**Figure 2:**
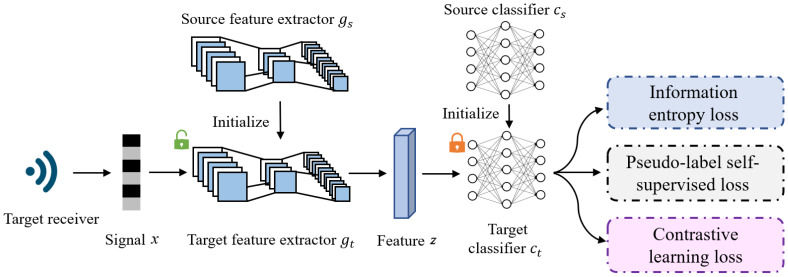
The overview of proposed method CSCNet. The model comprises two primary components: the feature extractor (*g*) and the classifier (*C*). The role of the feature extractor *g* is to extract emitter’s fingerprint from the received signal, while the classifier *C* maps the feature to the corresponding class. Our method involves loading the source model $g_{s}$ and $C_{s}$, which are pre-trained on the source domain $D_{s}$. Then, we freeze the parameters of $C_{s}$ and update the feature extractor *g* to adapt the target domain $D_{t}$. Three kinds of losses are considered, namely, information entropy loss, pseudo-label self-supervised loss, and contrastive learning loss.

The CSCNet, as depicted in Figure 2, is structured to address the adaptation challenge. The model $h:X\to \Delta_{K}$ integrates a feature extractor $g\left(\cdot ,\theta_{F}\right):X\to R^{d}$ and a classifier $C\left(\cdot ,\theta_{C}\right):R^{d}\to \Delta_{K}$, formulated as h(x)=C∘g(x). Here, X denotes the signal space, *d* represents the dimensionality of the feature space, and $\theta_{F}$ and $\theta_{C}$ are the respective parameters governing the feature extractor and classifier.

At its core, the CSCNet model is composed of two primary, interconnected components: a feature extractor (*g*) and a classifier (*C*). The feature extractor’s crucial role is to meticulously extract the unique radio frequency fingerprints from the received signals. These fingerprints, which are subtle characteristics stemming from inherent hardware imperfections, are then passed to the classifier. The classifier’s function is to accurately map these extracted features to their corresponding emitter class, thereby identifying the transmitting device. Our method involves loading the source model $g_{s}$ and $C_{s}$, which are pre-trained on the source domain $D_{s}$. Subsequently, we freeze the parameters of $C_{s}$ and update the feature extractor *g* to adapt to the target domain $D_{t}$.

The adaptation strategy of CSCNet involves leveraging a trained source model $h_{s}=C_{s}\circ g_{s}$, which has been trained using supervised learning on the labeled source domain $D_{s}$ to minimize cross-entropy loss. In the adaptation phase to the target domain $D_{t}$, the classifier’s parameters $\theta_{C}$ remain fixed, while the feature extractor’s parameters $\theta_{F}$ are re-optimized to better align with the characteristics of $D_{t}$. According to Theorem 1, solving problem (2) is theoretically beneficial for achieving domain adaptation. However, due to the inherent constraints in problem (2), we explore an alternative approach by minimizing its penalized form:

(9) $$\min_{\theta_{F}}L\left(\theta_{F}\right)≜\hat{\epsilon }\left(h,{\tilde{f}}_{t}\right)+\lambda L^{\mathrm{entropy}}\left(\theta_{F}\right),$$

where $\hat{\epsilon }\left(h,{\tilde{f}}_{t}\right)$ denotes the empirical risk with respect to the pseudo-label function ${\tilde{f}}_{t}$. Here, $L^{\mathrm{entropy}}\left(\theta_{F}\right)$ represents a regularization term to promote generalization, controlled by the hyperparameter λ.

Specifically, CSCNet integrates a three-pronged loss function strategy to achieve robust model adaptation. This strategy includes minimizing information entropy loss to encourage confident predictions, implementing pseudo-label self-supervised loss to leverage self-generated labels for training, and crucially, incorporating contrastive learning loss to capture the intricate relationships between signal samples effectively. This multi-faceted approach allows the model to learn powerful representations solely from unlabeled data on the deployed receiver, significantly enhancing recognition accuracy and robustness in cross-receiver RFFI scenarios.

The optimization of problem (9) is intricately linked to the pseudo-label function ${\tilde{f}}_{t}$, and vice versa. However, jointly optimizing ${\tilde{f}}_{t}$ and $\theta_{F}$ can be challenging. To address this, an alternating optimization strategy is proposed:

1. **Initialization:** Begin with ${\tilde{f}}_{t}$ initialized using the source-trained model $h_{s}$.
2. **Pseudo-Label Generation:** Utilize ${\tilde{f}}_{t}$ to generate pseudo-labels ${\tilde{y}}_{t}$ for all samples $x_{t}\in D_{t}$.
3. **Feature Extractor Update:** Formulate and solve problem (9) using the generated pseudo-labels ${\tilde{y}}_{t}$ to update $\theta_{F}$.
4. **Pseudo-Label Update:** Update the pseudo-label function ${\tilde{f}}_{t}$ with the latest network parameters: ${\tilde{f}}_{t}\leftarrow h\left(\cdot ;\theta_{F}\right)$.
5. **Iteration:** Repeat steps (2)–(4) iteratively until convergence criteria are met.

These iterative steps are crucial for progressively refining the feature extractor *g* to better capture domain-specific characteristics while continuously updating the pseudo-label function ${\tilde{f}}_{t}$ to improve model adaptation. Subsequent subsections will delve deeper into each of these steps, providing comprehensive insights and experimental validations to support the proposed approach.

### 4.1. Pseudo-Label Generation

The source-trained model $h_{s}$ provides an initial basis for generating pseudo-labels in the target domain $D_{t}$. For each target data point $x_{t}\in D_{t}$, the pseudo-label ${\tilde{y}}_{t}$ is determined as

$${\tilde{y}}_{t}=\arg\max_{k\in \{1,\ldots ,K\}}{\left[h_{s}\left(x_{t}\right)\right]}_{k},$$

where ${\left[h_{s}\left(x_{t}\right)\right]}_{k}$ denotes the *k*-th element of the output vector $h_{s}\left(x_{t}\right)$, representing the model’s confidence that $x_{t}$ belongs to class *k*.

However, due to the domain shift between $D_{s}$ and $D_{t}$, these initial pseudo-labels ${\tilde{y}}_{t}$ are often not accurate and can be sensitive to domain discrepancies. To improve their accuracy, we propose a clustering-based pseudo-labeling strategy:

1. **Initialization:** Start with ${\tilde{f}}_{t}\leftarrow h_{s}$, where ${\tilde{f}}_{t}\left(x\right)$ denotes the output of the source-trained model $h_{s}$ for signal *x*.
2. **Initial Weighted Center Calculation:** Compute the initial weighted center $c_{k}$ for each class *k* using the feature vectors $g(x_{t},\theta_{F})$ extracted by the feature extractor $g(\cdot ,\theta_{F})$:
  $$c_{k}=\frac{\sum_{x_{t}\in D_{t}}{\left[{\tilde{f}}_{t}\left(x_{t}\right)\right]}_{k}\cdot g\left(x_{t},\theta_{F}\right)}{\sum_{x_{t}\in D_{t}}{\left[{\tilde{f}}_{t}\left(x_{t}\right)\right]}_{k}},\;k=1,\ldots ,K.$$
  Here, ${\left[{\tilde{f}}_{t}\left(x_{t}\right)\right]}_{k}$ denotes the *k*-th component of ${\tilde{f}}_{t}\left(x_{t}\right)$.
3. **Pseudo-Label Assignment:** Assign pseudo-labels ${\tilde{y}}_{t}$ to each target data point $x_{t}\in D_{t}$ based on cosine similarity with the class centers $c_{k}$:
  $${\tilde{y}}_{t}=\arg\max_{k\in \{1,\ldots ,K\}}\frac{\langle {\tilde{f}}_{t}\left(x_{t}\right),c_{k}\rangle }{∥{\tilde{f}}_{t}\left(x_{t}\right)∥\cdot ∥c_{k}∥},$$
  This step ensures that ${\tilde{y}}_{t}$ is assigned to the class whose centroid $c_{k}$ is most similar to ${\tilde{f}}_{t}\left(x_{t}\right)$.
4. **Update of Target Feature Centers:** Update the class centers $c_{k}$ using k-means clustering with the updated pseudo-labels ${\tilde{y}}_{t}$:
  $$c_{k}=\frac{\sum_{x_{t}\in D_{t}}1_{{\tilde{y}}_{t}=k}\cdot g\left(x_{t},\theta_{F}\right)}{\sum_{x_{t}\in D_{t}}1_{{\tilde{y}}_{t}=k}},\;k=1,\ldots ,K.$$
  This step adjusts $c_{k}$ by averaging the feature vectors of data points $x_{t}$ assigned to class *k*, ensuring the centers reflect the current distribution of the target domain.
5. **Iteration:** Iterate steps (3) and (4) until convergence criteria are met for the k-means algorithm. Convergence indicates stability in the assignment of pseudo-labels ${\tilde{y}}_{t}$ and the centroids $c_{k}$.

By iteratively refining the pseudo-labels based on clustering with the feature vectors and updating the class centers accordingly, this approach progressively improves the alignment of the model *h* with the target domain $D_{t}$. This method effectively mitigates the domain shift between $D_{s}$ and $D_{t}$, enhancing the adaptation process of the model for accurate classification on the target domain.

### 4.2. Feature Extractor Update

Given the pseudo-label ${\tilde{y}}_{t}$ assigned to each target data point $x_{t}\in D_{t}$, we utilize standard supervised learning techniques to update the parameters $\theta_{F}$ of the feature extractor. Instead of using the empirical risk $\hat{\epsilon }$ defined in Equation (9), we adopt the cross-entropy loss, which measures the discrepancy between predicted probabilities and the pseudo-labels:

$$L^{\mathrm{CE}}\left(\theta_{F}\right)≜-\frac{1}{N_{t}}\sum_{i=1}^{N_{t}}\sum_{k=1}^{K}1_{{\tilde{y}}_{t}^{i}=k}\cdot \log{\left[h\left(x_{t}^{i};\theta_{F}\right)\right]}_{k},$$

where $h(x_{t};\theta_{F})$ denotes the output probability vector of the classifier *C* with parameters $\theta_{F}$.

To further enhance the utilization of pseudo-label information, we employ contrastive learning techniques, inspired by recent advancements in representation learning [15]. Contrastive learning aims to improve the feature representation by encouraging similar data points to be closer together and dissimilar ones to be farther apart in the feature space. The contrastive learning loss function $L^{\mathrm{CL}}\left(\theta_{F}\right)$ is designed to maximize the agreement between the feature representations of instances sharing the same pseudo-label and differentiate those from different classes. It is defined as

$$L^{\mathrm{CL}}\left(\theta_{F}\right)=-\frac{1}{N_{t}}\sum_{i=1}^{N_{t}}\sum_{k=1}^{K}1_{{\tilde{y}}_{t}^{i}=k}\cdot \log\frac{\exp\left[{\mathrm{sim}}_{k}/\tau \right]}{\sum_{j=1}^{K}\exp\left[{\mathrm{sim}}_{j}/\tau \right]},$$

where τ>0 is a temperature hyper-parameter controlling the sharpness of the softmax function, and ${\mathrm{sim}}_{k}$ represents the cosine similarity between the feature vector $g(x_{t}^{i},\theta_{F})$ and a learnable vector $w_{k}$ associated with the *k*-th class:

$${\mathrm{sim}}_{k}=\frac{\langle w_{k},g\left(x_{t}^{i},\theta_{F}\right)\rangle }{∥w_{k}∥∥g\left(x_{t}^{i},\theta_{F}\right)∥}.$$

Here, $w_{k}$ corresponds to the parameters of the fully connected layer in the classifier *C*. It should be noted that since the classifier *C* is frozen, the $w_{k}$ is fixed and not updated during adaptation.

In summary, the parameter $\theta_{F}$ of the feature extractor is updated by minimizing the combined loss:

$$\min_{\theta_{F}}\tilde{L}\left(\theta_{F}\right)≜L^{\mathrm{CE}}\left(\theta_{F}\right)+\beta L^{\mathrm{CL}}\left(\theta_{F}\right)+\lambda L^{\mathrm{entropy}}\left(\theta_{F}\right),$$

where β and λ are hyper-parameters controlling the contributions of contrastive learning and entropy regularization, respectively. The update of $\theta_{F}$ using gradient descent is performed iteratively:

$$\theta_{F}\leftarrow \theta_{F}-\alpha \frac{\partial \tilde{L}\left(\theta_{F}\right)}{\partial \theta_{F}},$$

where α>0 denotes the learning rate.

This approach integrates supervised learning with cross-entropy loss and unsupervised contrastive learning to adapt the feature representation $g(\cdot ,\theta_{F})$ to the target domain $D_{t}$. By leveraging pseudo-labels and contrastive learning, the model effectively learns a discriminative feature space that mitigates domain shift challenges, enhancing its performance in target domain tasks.

## 5. Experiment

### 5.1. Setups

In this section, we will evaluate the performance of our model using two real-world datasets: HackRF and Wisig [16]. The HackRF dataset comprises data received from multiple HackRF hardware devices. On the other hand, Wisig is a large-scale WiFi dataset, and for our experiment, we use a subset of Wisig, called “ManySig”. The datasets consist of signals received by multiple receivers, from which we can choose two. One receiver is designated as the source receiver, and the other is the target receiver. In the following subsections, we provide a brief overview of the datasets and describe the implementation of our method.

#### 5.1.1. Dataset

**Figure 3:**
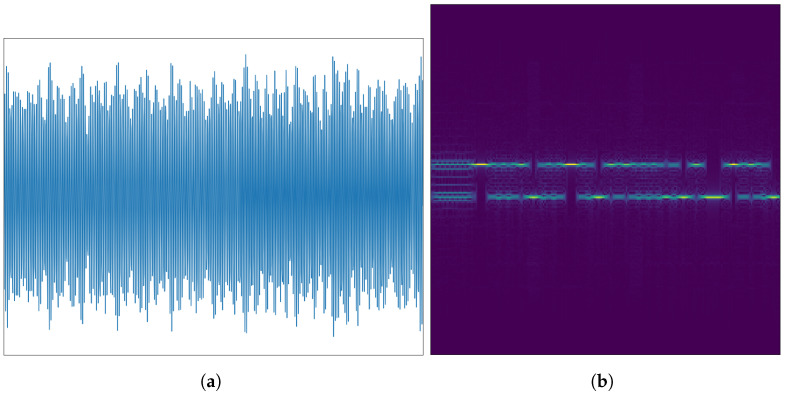
Signals of HackRF dataset: (**a**) waveform of HackRF; (**b**) spectrogram of HackRF.

HackRF, a cost-effective open-source software radio platform, is used to generate and collect the HackRF dataset, which includes signal data. Data collection involved four HackRFs as transmitting terminal devices and three HackRFs (referred to as HackRF 1, 2, and 3) as receivers. Signals were generated using Matlab and modulated using BFSK with a 2 MHz sampling rate. To introduce randomness, amplitude and frequency noise were uniformly added to the signals. This process ensures the dataset simulates realistic communication environments where signal quality can be degraded, thereby providing a basis for evaluating the model’s robustness against noise. The transmitted signals passed through a relatively fixed channel and were moved to the intermediate frequency (IF) before being received by the receiving device. The collected signals were transformed into signal time-domain samples using data frames, with each time-domain sample containing 28,000 sampling points. By performing short-time Fourier transform (STFT) on each time-domain signal sample, the corresponding spectrogram is obtained. The time-domain signal and its spectrum are illustrated in Figure 3.

**Figure 4:**
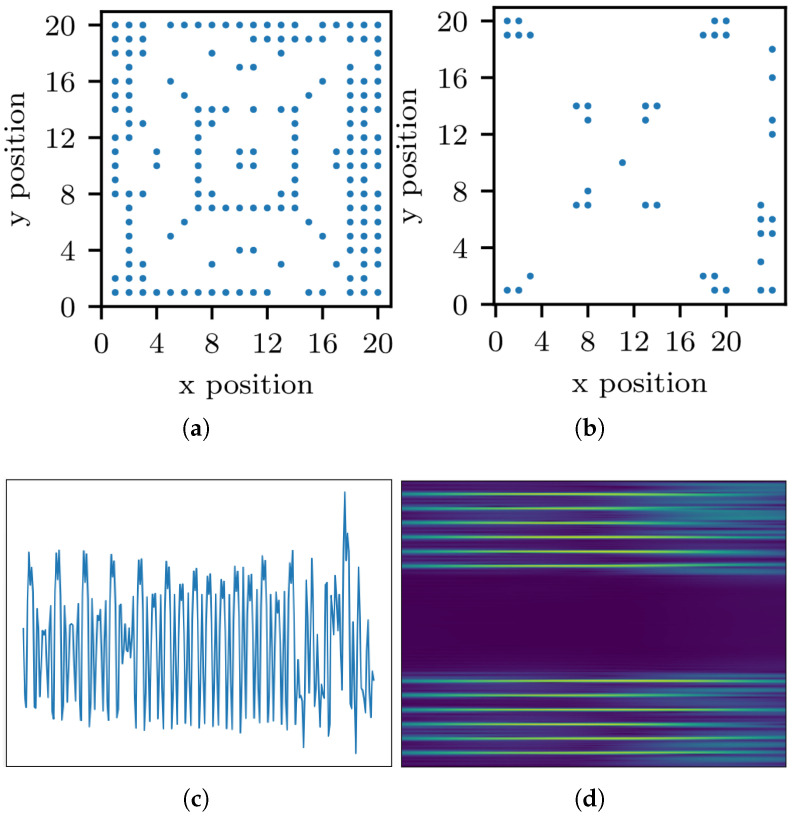
Scenario description of Wisig dataset [16]: (**a**) positions of Tx; (**b**) positions of Rx; (**c**) waveform of Wisig; (**d**) spectrogram of Wisig.

The Wisig dataset [16] is a large-scale WiFi dataset containing 10 million packets obtained from 174 off-the-shelf WiFi transmitters (Tx) and 41 USRP receivers (Rx) across four captures over a month. The Tx and Rx are deployed on nodes arranged in a grid. The 2D-coordinate scattergram of Tx and Rx is shown in Figure 4a,b, respectively. For our experiment, we used the ManySig subset provided by the dataset, comprising 1000 equalized signals from all Tx-Rx pairs, including 6 Tx and 12 Rx, spanning over four days. As a large-scale dataset captured in a real-world environment, its signals inherently encompass a range of channel effects and authentic signal-to-noise ratios, offering a practical benchmark for the model’s performance under non-ideal conditions. The time-domain signal and its spectrum of Wisig dataset are illustrated in Figure 4c,d. Each signal contains two channels and 256 sample points. During the cross-receiver test, the dataset is divided into 12 parts, each corresponding to a receiver with capturing time spanning over four days. The time-domain signal and its spectrum of Wisig dataset are illustrated in Figure 4c,d.

#### 5.1.2. Implementation Details

The feature extractor for HackRF employs ResNet50 [17], while for Wisig, ResNet18-1D is utilized. To adapt to one-dimensional time series signals, ResNet18-1D is derived from ResNet18 by substituting the 2D-convolution with 1D-convolution. Additionally, the classifier networks are implemented using a four-layer fully connected network. For data preprocessing, we applied min-max normalization to scale the RF samples to the range of [−1,1]. This normalization aids in the convergence of the neural network training. The formula used for this normalization is

$$Z_{min-max}=\frac{2(z-Z_{min})}{Z_{max}-Z_{min}}-1$$

where *z* represents the input data, $Z_{min-max}$ is the normalized output, and $Z_{min}$ and $Z_{max}$ are the minimum and maximum values of *z*, respectively. No additional data augmentation or filtering methods were applied to the raw RF signals beyond this normalization.

The initial learning rate is set as 0.0001. The hyperparameters λ and β are set to 0.2 and 0.5, respectively. The temperature coefficient τ=0.1, and the total number of training epochs is set to 50. As for the final results, we calculate the classification accuracies of the models on the test dataset for the last five epochs of each experiment.

### 5.2. Comparison with the Source-Only Method

To assess the effectiveness of our network model, we conducted a comparison with source-only methods. The source-only method utilizes only cross-entropy loss as the loss function and trains the network solely on data from the source receiver, without employing any other algorithms for adaptation.

Table 1 presents the performance comparison between the traditional method and our proposed approach when using different datasets. In the HackRF dataset, the *i*-th receiver is called “HackRF *i*”, while “HackRFi→HackRFj” denotes adaptation from receiver HackRF *i* to HackRF *j*. We designate HackRF *i* as the source receiver, while HackRF *j* serves as the target receiver. For the Wisig dataset, to uniquely represent transmitters (Tx) and receivers (Rx) on a 2D grid, we assign them two-dimensional (2D) coordinates, denoted as “x-y” for receiver located at (x,y). The notation “$x-y\to x^{\prime }-y^{\prime }$” signifies adaptation from data collected at receiver “x-y” to that of “$x^{\prime }-y^{\prime }$”. Similar to the HackRF dataset, we designate the receiver “x-y” as the source receiver, and the receiver “$x^{\prime }-y^{\prime }$” as the target receiver.

Upon testing, when the same receiver is applied for training and testing, the recognition accuracies of the source-only model attained 93.77% and 99.99% for the HackRF dataset and the Wisig dataset, respectively. Therefore, for the within-receiver test, the source-only model already works very well. However, as shown in Table 1, when the training and test data come from different receivers, the source-only model’s recognition accuracy dramatically drops to around 50% to 60%, indicating the significant impact of the receiver’s fingerprint on the results. In contrast, our proposed CSCNet model achieves significantly improved recognition accuracy, with an improvement of at least 13%. Notably, in the “1-19 → 7-7” task, the recognition accuracy improved by 47%. These results conclusively demonstrate the effectiveness of the CSCNet model in addressing the receiving impact.

### 5.3. Comparison with Other Domain Adaptation Methods

We conducted a comparison of our proposed method with other domain adaptive methods on the Wisig dataset, including DANN [18], MCD [19], and SHOT [20].

As presented in Table 2, our CSCNet method outperforms the other domain-adaptive methods significantly. In comparison with the best-performing method, CSCNet demonstrates a remarkable improvement in recognition accuracy across different tasks. For the task “1-1 → 1-19”, CSCNet achieves an accuracy of 92.64%, which is significantly higher than the best alternative method, MCD, which has an accuracy of 79.64%. For the task “1-1 → 8-8”, although DANN shows a slightly higher accuracy of 96.52%, CSCNet still performs competitively with an accuracy of 95.72%. In the task “7-7 → 8-8”, CSCNet achieves the highest accuracy of 88.00%, surpassing all other methods. This demonstrates the robustness and effectiveness of CSCNet in various domain adaptation scenarios.

### 5.4. Ablation Experiment

We conducted a series of ablation experiments to evaluate the components of our proposed method, specifically focusing on two key elements: the information entropy loss (IEL) and the contrastive learning loss (CLL). To isolate the effects of each component, we designed four configurations: a baseline with no losses, serving as a control to measure the performance impact of each loss; a setup incorporating only the IEL; another configuration employing only the CLL; and a final setup combining both losses to explore their synergistic effects on model performance. Extensive experiments were carried out across multiple cross-domain tasks, with the results summarized in Table 3, demonstrating the individual and combined contributions of each component to the overall effectiveness of the method.

As shown in Table 3, the baseline configuration, which does not include any specific loss functions, achieves accuracies of 59.14%, 62.42%, and 44.50% for the tasks “1-1 → 1-19”, “1-1 → 8-8”, and “7-7 → 8-8”, respectively. When only the information entropy loss (IEL) is applied, the performance improves significantly, with accuracies reaching 74.61%, 88.73%, and 74.73% for the same tasks. On the other hand, the configuration using only the contrastive learning loss (CLL) shows a decline in performance, achieving accuracies of 49.38%, 49.15%, and 39.81%. The most notable improvement is observed when both IEL and CLL are combined, resulting in the highest accuracies of 92.64%, 95.72%, and 88.00%, indicating a strong synergistic effect between these two components.

### 5.5. Effect of SNR on Model Accuracy

**Figure 5:**
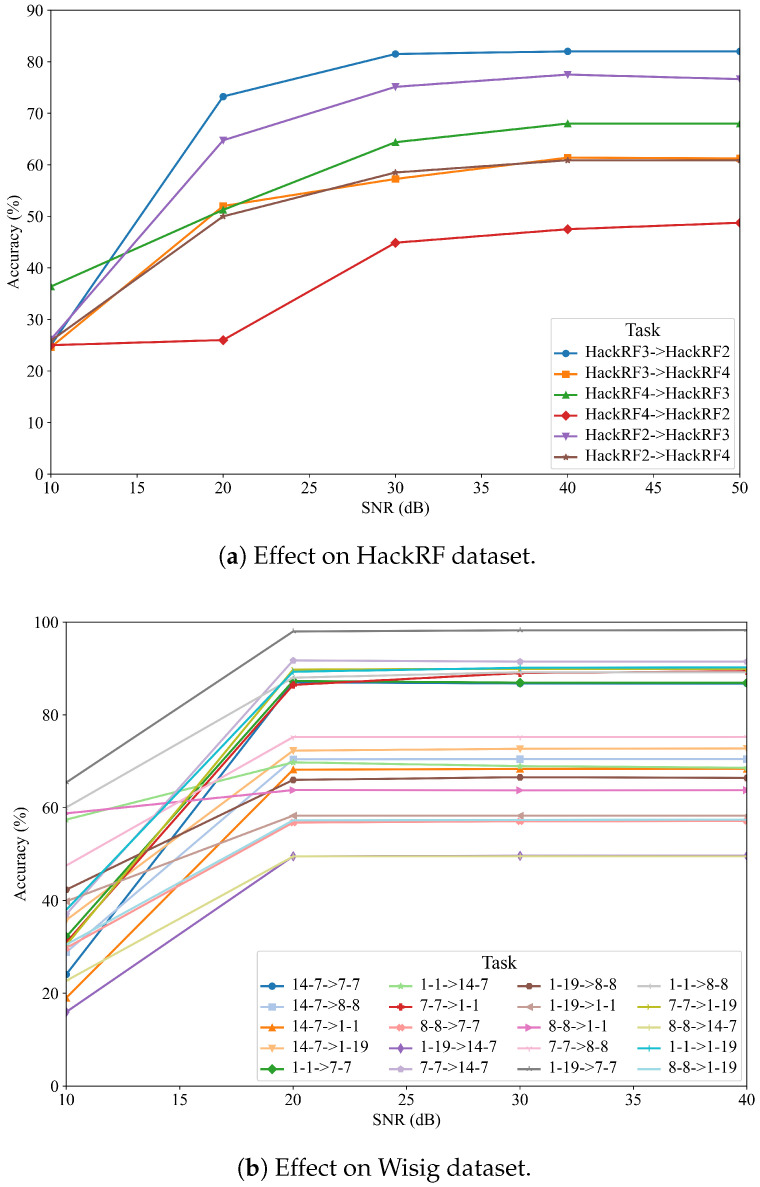
Effect of SNR on the accuracy of CSCNet for various cross-receiver tasks on the (**a**) HackRF and (**b**) Wisig datasets.

To address the practical deployment of the model and evaluate its robustness, we investigated the effect of the signal-to-noise ratio (SNR) on the accuracy of CSCNet. We simulated various noise conditions by adding additive white Gaussian noise (AWGN) to the test signals of both the HackRF and Wisig datasets to achieve SNR levels of 0 dB, 5 dB, 10 dB, 15 dB, and 20 dB. The results are presented for several cross-receiver adaptation tasks in Figure 5.

Figure 5a illustrates the performance of CSCNet on the HackRF dataset under different SNR conditions. As expected, the model’s accuracy is significantly impacted by noise. At a low SNR of 0 dB, the performance is generally poor across all tasks, with accuracies ranging from 25% to 36%. However, as the SNR increases to 5 dB and 10 dB, there is a substantial improvement in accuracy. For instance, in the ‘h2 → h3’ task, the accuracy jumps from 26.12% at 0 dB to 75.12% at 10 dB. For most tasks, the performance tends to saturate or plateau when the SNR is 10 dB or higher, indicating that the model performs robustly in moderately noisy environments.

Similarly, Figure 5b shows the results for the Wisig dataset. The trend is consistent, where higher SNR leads to better performance. The influence of noise is particularly evident in challenging adaptation tasks. For example, in the ‘1-19 → 14-7’ task, the accuracy is only 15.96% at 0 dB but climbs to nearly 50% at 10 dB. In contrast, for tasks where the model adapts well, such as ‘1-19 → 7-7’, the accuracy starts at a respectable 65.42% at 0 dB and quickly reaches over 98% at higher SNRs. This analysis confirms that while CSCNet’s performance is dependent on signal quality, it demonstrates strong robustness and achieves high accuracy under realistic noise conditions (SNR ≥ 10 dB), which is crucial for real-world applications. These results also underscore that some cross-receiver pairs are inherently more challenging to adapt to, regardless of the noise level.

### 5.6. t-SNE Visualization

**Figure 6:**
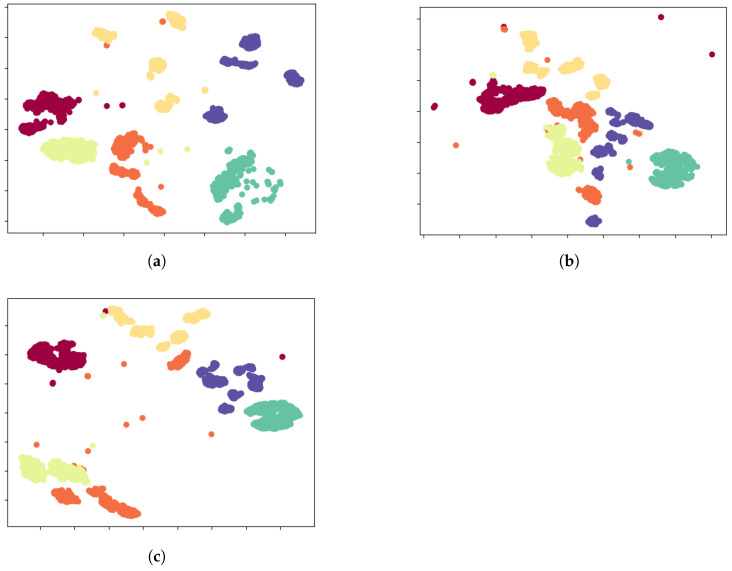
t-SNE visualization of the learned feature representations, providing a qualitative assessment of the model’s effectiveness. In all subplots, different colors are used to distinguish signals from different transmitter classes. The clustering of same-colored points and the separation between different-colored clusters visually represent the model’s classification capability. (**a**) Features from the source domain data, extracted by the source-trained model, show clear and well-separated class clusters, representing the ideal performance baseline. (**b**) When the source model is applied directly to the target domain data, the feature representations of different classes (colors) become heavily overlapped and indistinguishable, visually demonstrating the severe impact of the domain shift problem. (**c**) Features from the target domain data, extracted by our proposed CSCNet model, show that the class-specific clusters (colors) are reformed and become significantly more separable, which powerfully demonstrates the effectiveness of our source-free adaptation method.

To provide compelling qualitative validation for our method, we employed t-SNE to visualize the learned high-dimensional feature representations, with the results depicted in Figure 6. This visualization offers a clear narrative of the problem and our solution’s efficacy. Initially, for the in-domain scenario, the source-trained model demonstrates its full potential by mapping source data into a well-structured feature space, characterized by high intra-class compactness and clear inter-class separation (Figure 6a). This ideal clustering signifies that the learned features are highly discriminative. However, this finely-tuned feature structure proves fragile when confronted with the cross-receiver domain shift. When the model is applied to target domain data, the feature space integrity collapses into a chaotic and non-discriminative manifold where features from different classes become severely entangled (Figure 6b). This visual evidence explains why traditional classifiers fail: No simple decision boundary can effectively partition such an overlapped space. This is the critical challenge CSCNet addresses. Through its source-free adaptation strategy—which uniquely combines information entropy minimization with pseudo-label-guided contrastive learning—CSCNet actively remodels the target feature space. It learns to disentangle the mixed representations by pulling samples with the same pseudo-label closer together while pushing others apart. The result is a remarkable restoration of order, as seen in Figure 6c, where distinct, albeit not as perfect as the source-domain baseline, clusters re-emerge. This visual journey from a well-ordered space, through a chaotic collapse, to a successfully restructured state powerfully demonstrates CSCNet’s ability to learn domain-agnostic features. It serves as strong corroborating evidence for the quantitative accuracy gains, confirming that the performance improvement stems from a fundamental enhancement of the feature representation itself.

## 6. Conclusions

This paper introduced the source-free cross-receiver rf fingerprinting (SCRFFI) problem, tackling the crucial challenge of adapting pre-trained RF fingerprinting models to new receivers without accessing original training data, a vital aspect for data privacy and deployment. Our theoretical analysis provided an upper bound on generalization performance, emphasizing the role of pseudo-label accuracy. To address this, we proposed CSCNet, a novel approach leveraging information entropy loss, pseudo-label self-supervised loss, and contrastive learning loss. Experimental results on HackRF and Wisig datasets demonstrated CSCNet’s significant effectiveness. It consistently outperformed the source-only method, with recognition accuracy improvements of at least 13% and, notably, a 47% gain in the “1-19→7-7” task on Wisig (from 50.88% to 98.12%). CSCNet also surpassed other domain adaptation methods like DANN, MCD, and SHOT. Ablation studies confirmed the synergistic contribution of information entropy and contrastive learning losses. These achievements highlight CSCNet’s robustness in mitigating receiver variations and its practical value for RFFI deployment.

## Figures and Tables

**Table 1:** Classification accuracies (%) comparison with source-only method.

Task	Method		Improvement
	Source-Only	CSCNet	
HackRF 1 → HackRF 2	50.74	75.33	24.59
HackRF 1 → HackRF 3	41.74	68.29	26.55
HackRF 2 → HackRF 1	32.95	81.13	48.36
HackRF 3 → HackRF 1	29.71	53.21	23.5
1-1 → 1-19	59.14	92.64	33.50
1-1 → 7-7	74.09	86.93	12.83
1-1 → 8-8	67.21	95.72	29.17
1-19 → 7-7	50.88	98.12	47.24
14-7 → 7-7	52.60	86.78	34.18
7-7 → 14-7	55.80	91.47	35.66
7-7 → 1-1	68.24	89.03	20.78
7-7 → 1-19	58.46	89.80	31.34
8-8 → 1-19	57.41	70.35	12.94
8-8 → 14-7	49.52	67.06	17.53

**Table 2:** Classification accuracies (%) comparison with other UDA methods.

Method	1-1 → 1-19	1-1 → 8-8	7-7 → 8-8
DANN [18]	74.82	**96.52**	70.32
MCD [19]	79.64	62.75	66.95
SHOT [20]	79.37	83.21	66.72
CSCNet (proposed)	**92.64**	95.72	**88.00**

**Table 3:** Ablation accuracies (%).

Method	1-1 → 1-19	1-1 → 8-8	7-7 → 8-8
Baseline	59.14	62.42	44.50
CLL	49.38	49.15	39.81
IEL	74.61	88.73	74.73
IEL+CLL	**92.64**	**95.72**	**88.00**

## Data Availability

Data will be made available on request.
